# A study of optimal concentration range and time window of sevoflurane preconditioning for brain protection in MCAO rats

**DOI:** 10.1186/s12871-020-00984-1

**Published:** 2020-04-05

**Authors:** Ying Deng, Chengmei Shi, Yi Gu, Ning Yang, Mao Xu, Ting Xu, Xiangyang Guo

**Affiliations:** 1 Department of Anesthesiology, Peking University Third Hospital, No. 49, North Garden Street, Haidian District, Beijing, 100191 China; 2 Beijing Tiantan Hospital, Capital Medical University, No. 119 South 4th Ring West Road, Fengtai District, Beijing, 100160 China

**Keywords:** Sevoflurane, Preconditioning, Cerebral protection, Concentration range, Time window

## Abstract

**Background:**

Sevoflurane preconditioning improves brain function in MCAO rats, and there are several methods for determining appropriate concentration and time windows for preconditioning. This study investigated the brain protective effects with a single sevoflurane preconditioning at different concentrations and different time windows on MCAO rats.

**Methods:**

Adult Sprague-dawley rats were randomly assigned to 14 groups. The rats in the sevoflurane preconditioning group inhaled 0.5 MAC, 1.0 MAC, and 1.3 MAC sevoflurane, respectively for 3 h, and then MCAO models were established at 6 h, 12 h, 24 h, and 48 h. MCAO and sham groups underwent no preconditioning with sevoflurane. The neurological severity score, cerebral infarct volume and brain water content of the rats were measured 24 h after reperfusion.

**Results:**

After inhalation of 1.3 MAC sevoflurane for 3 h of preconditioning, the MCAO model was established after 24 h. This preconditioning improved the neurological severity score, reduce cerebral infarct volume and brain water content in MCAO rats. After inhalation of 1.0 MAC sevoflurane for 3 h of preconditioning, MCAO model established after 24 h reduced the cerebral infarct volume and brain water content of MCAO rats, but the neurological severity score showed no significant improvement, and no significant brain protective effects were observed at other concentrations and time windows.

**Conclusions:**

These results suggested that after inhalation of 1.3 MAC sevoflurane for 3 h of preconditioning, MCAO model established after 24 h demonstrated significant brain protective effects in MCAO rats.

## Background

Stroke is the leading cause of disability [1] and is a major cause of morbidity [2], leading to high economic burden worldwide. About 30% patients die directly due to the disease each year, and similar percentage of sufferers are functionally disabled [2, 3].

Ischemic stroke is more commonly seen and is caused by the embolization or thrombosis in blood vessels of the brain, resulting in energy metabolism disorders, ionic homeostasis imbalances and free radical and excitatory neurotoxicity production [4, 5]. Especially for perioperative ischemic stroke patients, despite its low morbidity [6], it is regarded as a catastrophic event that affects the prognosis and outcomes of surgery.

Currently, the most useful strategy for stroke mainly depends on early detection and thrombolytic therapy [1, 7]. However, due to very narrow time window for anticoagulant therapy, many patients miss the best time for effective treatment. Therefore, several recent studies have focused on neuroprotection strategies of stroke [8, 9]. These therapeutic approaches have important clinical and social significances in effectively studying the prevention and treatment measures of perioperative stroke.

Preconditioning is an important biological phenomenon that often occurs under non-lethal conditions, such as high temperatures, exercise, systemic or ischemia, inflammation or general anesthesia [9, 10]. This is defined as delivering a stimulus below the threshold for tissues or organs to develop tolerance to induce subsequent stimuli above the threshold. Preconditioning measures included volatile inhalational anesthetics, hypoxia, ischemia, cortical spreading inhibition, and pro-inflammatory drugs [11]. Preconditioning can induce several internal signaling pathways to protect the body from more severe ischemic damage [12]. Preconditioning with volatile anesthetics induces tolerance to cerebral ischemia/reperfusion injury in animals, and prevents neurologic complications such as perioperative stroke in patients [10]. Sevoflurane is one of the popular inhalational anesthetics that has neuroprotective properties during the perioperative period [11, 12]. Many studies have demonstrated the protective effects of sevoflurane against brain ischemia in vivo and in vitro [4, 13, 14]. Several studies have reported that sevoflurane preconditioning can inhibit oxygen free radicals [14], anti-inflammation [15], activate antioxidant enzymes [11], reduce the expression of apoptosis-related proteins [16], regulate TREK-1 and TREK- 2 channel [7, 8], and inhibit thioredoxin [17], thus reducing nerve injuries associated with middle cerebral artery occlusion (MCAO) in animals.

Current studies have not reached consensus regarding the appropriate concentration and time window of sevoflurane preconditioning. Hence, in the present study, sevoflurane preconditioning was performed at different concentrations and time windows to investigate appropriate concentration and time window regarding the protective effects of sevoflurane preconditioning in ischemia-reperfusion rats. We hypothesized that sevoflurane preconditioning could produce effective brain protective effects in MCAO rats wtih the optimal time window and concentration.

## Methods

### Animals

Male Sprague-Dawley (SD) rats (3 months of age, weighing 330–370 g) (Beijing VitalRiver Laboratory Animial Technology Co.China) were used for experiments in this study. Rats were bred and maintained under standardized housing connditions with food and water ad libitum. The experimental protocol was approved by the Peking University Biomedical Ethics Committee Experimental Animal Ethics Branch (Approval No. LA 2016300).

### Establishment of MCAO model

The MCAO model was established by using the suture method, and induced by sevoflurane inhalation. After anesthesia, a mask inhalation of 1 MAC sevoflurane + 60% oxygen was performed. During operation, the rectal temperature of the rats was maintained at 36–37 °C. After disinfecting neck, a median of 2 cm incision and separation were made, the distal right external carotid artery was ligated with a 4–0 surgical suture, and then a proximal slipknot ligation was performed. The common carotid artery and internal carotid artery were blocked with bulldog clamp. An oblique incision was made at the middle segment of the two ligation areas of external carotid artery, followed by the insertion of a thread (ZL400, Guangzhou Xinzan Biotechnology Co., Ltd.), and loosening of the bulldog clamp of the internal carotid artery. The thread was then inserted into the internal carotid artery, pushed to the initial segment of the anterior cerebral artery, and stopped until a resistance was reached, and it was generally not more than 20 mm. The blood supply to the middle cerebral artery was blocked, and the fixed thread for the suture was tightened, a bulldog clamp of the internal carotid artery was loosened, and then a suture was made to the wound. After 120 min, the thread that was exposed outside of the neck skin was gently pulled out and stopped until there was resistance. The excess parts were cut off to recirculate the blood, forming an ischemia-reperfusion model [18].

### Experimental methods

A total of 140 male SD rats were randomly assigned to 14 groups (10 in each group), which were as follows: 0.5 MAC (1%) sevoflurane 6 h, 12 h, 24 h, and 48 h group; 1.0 MAC (2%) sevoflurane 6 h, 12 h, 24 h, and 48 h group; 1.3 MAC (2.6%) sevoflurane 6 h, 12 h, 24 h, and 48 h group, MCAO group and sham group. Rats were preconditioned with sevoflurane for 3 h. MCAO and sham groups underwent no preconditioning with sevoflurane. The respiratory box was pre-filled with corresponding concentration of sevoflurane (sevoflurane + 60% oxygen) for 15 min. The rats were then placed in an anaesthetic respiratory box to maintain spontaneous breathing. The corresponding concentration of sevoflurane was continuously blown from the air inlet end, and the air outlet was connected to the atmosphere and the bypass end-expiratory sevoflurane concentration was detected to ensure that the MAC value remains unchanged at 1.3 MAC, 1.0 MAC, and 0.5 MAC. Rats in the sham and MCAO groups just inhaled a mixture of 60% oxygen and air for 3 h. Physiological parameters of the rats were monitored during preconditioning. The respiratory box was pre-filled with 60% oxygen, and the rats were placed in a respiratory box for 3 h. The respiratory box was evenly padded with a 37 °C insulation blanket to keep the rats warm. The MCAO model was established at different time periods (6 h/12 h/24 h/48 h) after preconditioning. There were 10 rats in each group (5 for measuring cerebral infarct volume and 5 for determining brain water content).

Except for these 140 rats, we took another 5 rats in each group of MCAO, Sham, 0.5 MAC, 1.0 MAC and 1.3 MAC respectively to determine whether sevflurane anesthesia caused physiologic side effects.

### Neurological severity scores (NSS) [3]

The neurological severity score was performed 24 h after MCAO operation in each group by blinding method (scoring was separately performed by two people and averaging). The NSS scoring standard was used with 18 points in total.

### The euthanasia

The rats were induced by sevoflurane inhalation. The respiratory box was pre-filled with 2% sevoflurane (sevoflurane + 60% oxygen) for 15 min. The rats were then placed in an anaesthetic respiratory box. The rats were immediately decapitated to take the brains When they were anesthetized and lost consciousness.

### Determination of cerebral infarct volume [19, 20]

After reperfusion for 24 h, the anesthetized rats with sevoflurane lost consciousness and were decapitated to take the brains, and the olfactory bulb, cerebellum and low brain stem were removed to make into sections. These sections were then placed in 20 ml of 2% triphenyltetrazolium chloride (TTC, Sigma) staining solution, followed by placing them in a water bath at 37 °C in dark place, and taken out after 10 min. Normal brain tissues appeared red and the infarct areas remained white. The brain sections were immediately kept in 4% paraformaldehyde for fixation for 24 h, and pictures were taken with a digital camera. The infarct volume of each section was calculated by using image analysis system Image ProPlus version 6.0. The percentage of cerebral infarct volume was calculated as the (total infarct volume of brain section /total area of​brain section) × 100%.

### Determination of brain water content [21]

Rats were anesthetized with sevoflurane, and immediately decapitated to remove the cerebellum and the lower brain stem. The micro-precision balance was immediately used to weigh the wet weight of the brain, placed in an oven, baked at 100 °C for 72 h until constant weight was reached. After weighing the dry weight, the brain water content was calculated by using the formula, water content (%) = (wet weight - dry weight)/wet weight × 100%.

### General pharmacology and pharmacokinetics

Sevoflurane is a new type inhalation anesthetic with quick inspiration, rapid induction and fine controllability. Generally associated with stable hemodynamics, dose dependent vasodilatation, and cardiac depression. And could also provide cardioprotection through pharmacologic preconditioning. Sevoflurane was administered through the lung and primarily eliminated by the lungs.

### Statistical analysis

We used Power and Sample Size 14.0 and superiority test method to calculate the sample size. Take α = 0.05, β = 0.9, according to the values in the literature, the sample size of each group was calculated to be 10.

Measurement data were expressed as mean ± standard deviation (x ± s). Statistical analysis was performed by using SPSS software, version 17.0. One-way analysis of variance was used for between-group comparison of data with normal distribution. The tukey was used for the post hoc analysis. The between-group comparison of the measurement data with skewed distribution was performed by using Kruskal and wallis method rank sum test, and the bonferroni adjusted method was used to modify the *p* value. Two-sided *P* < 0.05 was considered to be statistically significant.

## Results

### Effects of sevoflurane anesthesia on homeostasis of rats

After sevoflurane inhalation anesthesia, the righting reflex of the rats was disappeared within a few minutes, and the skin color of the nasolabial and toe ends of each group of rats appeared ruddy, with no significant fluctuations in SaO_2_, heart rate and rectal temperature. After anesthesia, the rats were awakened after 5–15 min.

To determine whether sevflurane anesthesia caused physiologic side effects, such as hypoxia, hypercapnia, or hypoglycemia, 5 rats were choosed in MCAO, Sham, 0.5 MAC, 1.0 MAC and 1.3 MAC respectively. MCAO and sham groups underwent no preconditioning with sevoflurane. 2 mL blood was withdrawn by cardiac puncture at the end of the sevflurane or oxygen exposure. In the MCAO group, 2 mL blood was withdrawn by cardiac puncture after the establishment of MCAO. Arterial blood gas (ABG) and blood glucose measurements were performed using a portable blood gas analyzer (OPTI Medical Systems, Georgia, USA) and One Touch Ultra blood glucose monitoring system (Life Scan Inc., California, USA) respectively. Those rats were not used for any other part of the study.

The results showed no statistical differences in pH, PaO_2_, PaCO_2_, and blood glucose for each group. No adverse reactions, such as hypoxemia, hypercapnia, and hypoglycemia were observed in each group. These results suggested that the effects of these adverse reactions on behavioral outcomes can be excluded (Table. 1).

**Table 1:** Effects of sevoflurane anesthesia on blood gas analysis in rats (*x* ± s, *n* = 5)

Group	pH	PaCO_2_ (mmHg)	PaO_2_ (mmHg)	Glucose (mmol/L)
MCAO	7.38 ± 0.06	37 ± 3	169 ± 6	4.9 ± 0.6
Sham	7.43 ± 0.04	40 ± 3	158 ± 10	5.1 ± 0.6
0.5 MAC	7.32 ± 0.03	38 ± 4	162 ± 9	5.2 ± 0.7
1.0 MAC	7.35 ± 0.05	36 ± 2	163 ± 7	5.0 ± 0.2
1.3 MAC	7.33 ± 0.04	35 ± 2	159 ± 8	5.1 ± 0.4

### Effects of sevoflurane anesthesia on behavioral function score

The behavioral score for the sham group was 0 (0–0), and that of the MCAO group was 11.5 (9.0–12.0). The behavioral score for 1.3 MAC sevoflurane preconditioning was 10.0 (7.0–12.0) for the 6 h group, 11.0 (10.0–13.0) for the 12 h group, 8.0 (6.0–9.0) for the 24 h group, and 11.0 (9.0–13.0) for the 48 h group. A statistically significant difference was observed between the MCAO group and 24 h group, but no statistical difference was observed for the comparison of 6 h, 12 h, and 48 h groups (*P* = 0.390, 0.809, 0.686).

The behavioral score for 1.0 MAC sevoflurane preconditioning was 11.0 (8.0–12.0) for the 6 h group, 11.0 (10.0–11.0) for the 12 h group, 9.5 (8.0–10.0) for the 24 h group, and 10.5 (8.0–11.0) for the 48 h group. There was no statistical differences between the MCAO group and the 6 h, 12 h, 24 h, and 48 h groups (*P* = 0.809, 0.882, 0.128, 0.420).

**Fig. 1:**
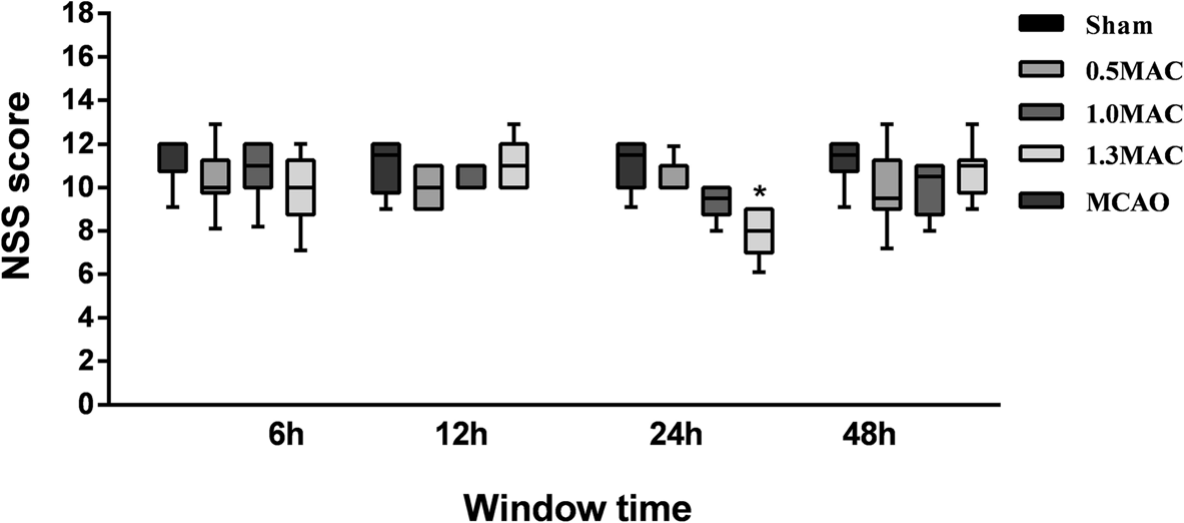
In 1.3 MAC sevoflurane preconditioning group, the behavioral score was significantly lower than the MCAO group, and showed no statistical difference between others groups and the MCAO group. Values are presented as mean (Lower-Upper limits), *n* = 10, *p < 0.05, vs. MCAO group

The behavioral score for 0.5 MAC sevoflurane preconditioning was 10.0 (8.0–13.0) for the 6 h group, 10.0 (9.0–11.0) for the 12 h group, 10.0 (10.0–12.0) for the 24 h group, and 9.5 (7.0–13.0) for the 48 h group. There was no statistical difference between the MCAO group and the 6 h, 12 h, 24 h, and 48 h groups (*P* = 0.809, 0.513, 0.809, 0.295), (Fig. 1).

### Comparison of cerebral infarct volume and percentage of infarct volume after MCAO

**Fig. 2:**
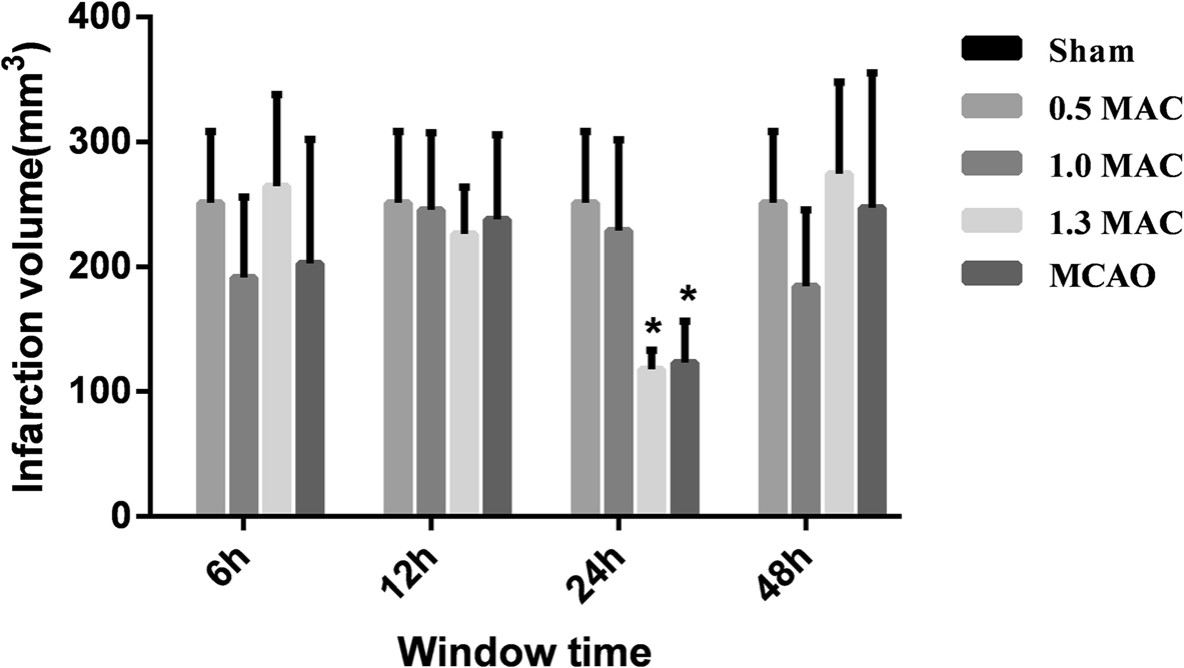
Comparison with MCAO group showed that the cerebral infarct volume was significantly smaller in 1.3 MAC sevoflurane preconditioning 24 h group. In 1.0 MAC sevoflurane preconditioning 24 h group, the cerebral infarct volume was significantly smaller than the MCAO group. There was no statistical difference between others groups and the MCAO group. Values are presented as mean ± SD, *n* = 5, *p < 0.05, vs. MCAO group

**Fig. 3:**
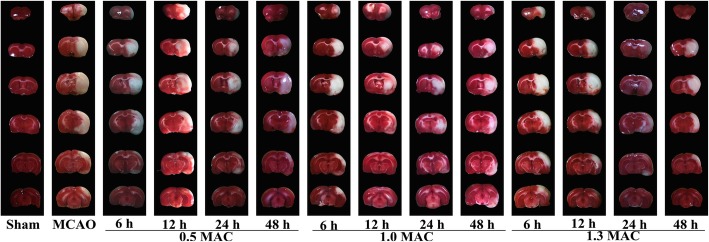
The red areas represent the normal brain tissues, and the white areas represent the infarction areas. In the 1.3 MAC sevoflurane preconditioning 24 h group and 1.0 MAC sevoflurane preconditioning 24 h group, the infarct areas were significantly smaller, especially in the 1.3 MAC sevoflurane preconditioning 24 h group

The cerebral infarct volume was 251.22 ± 57.32 mm^3^ in the MCAO group, 202.69 ± 99.34 mm^3^ in the 1.3 MAC sevoflurane preconditioning 6 h group, 237.87 ± 68.01 mm^3^ in the 12 h group, 123.11 ± 33.29 mm^3^ in the 24 h group, and 247.18 ± 107.91 mm^3^ in the 48 h group. There were statistical differences in the 24 h group (*P* = 0.002) when compared with MCAO group, while showed no statistical differences with 6 h, 12 h, and 48 h groups (*P* = 0.245, 0.747, 0.927). The cerebral infarct volume was 264.63 ± 73.52 mm^3^ in the 1.0 MAC sevoflurane preconditioning 6 h group, 226.17 ± 37.66 mm^3^ in the 12 h group, 117.68 ± 15.36 mm^3^ in the 24 h group, and 274.40 ± 73.49 mm^3^ in the 48 h group. When compared with MCAO group, the 24 h group (*P* = 0.004) showed statistical differences, while no statistical differences were observed with the 6 h, 12 h, and 48 h groups (*P* = 0.746, 0.605, 0.600). The cerebral infarct volume was 191.38 ± 64.58 mm^3^ in the 0.5 MAC sevoflurane preconditioning 6 h group, 245.36 ± 62.12 mm^3^ in the 12 h group, 228.95 ± 72.92 mm^3^ in the 24 h group, and 184.42 ± 61.02 mm^3^ in the 48 h group. There was no statistical difference with the 6 h, 12 h, 24 h, and 48 h groups as compared to the MCAO group (*P* = 0.180, 0.894, 0.591, 0.135), (Figs. 2 and 3).

**Fig. 4:**
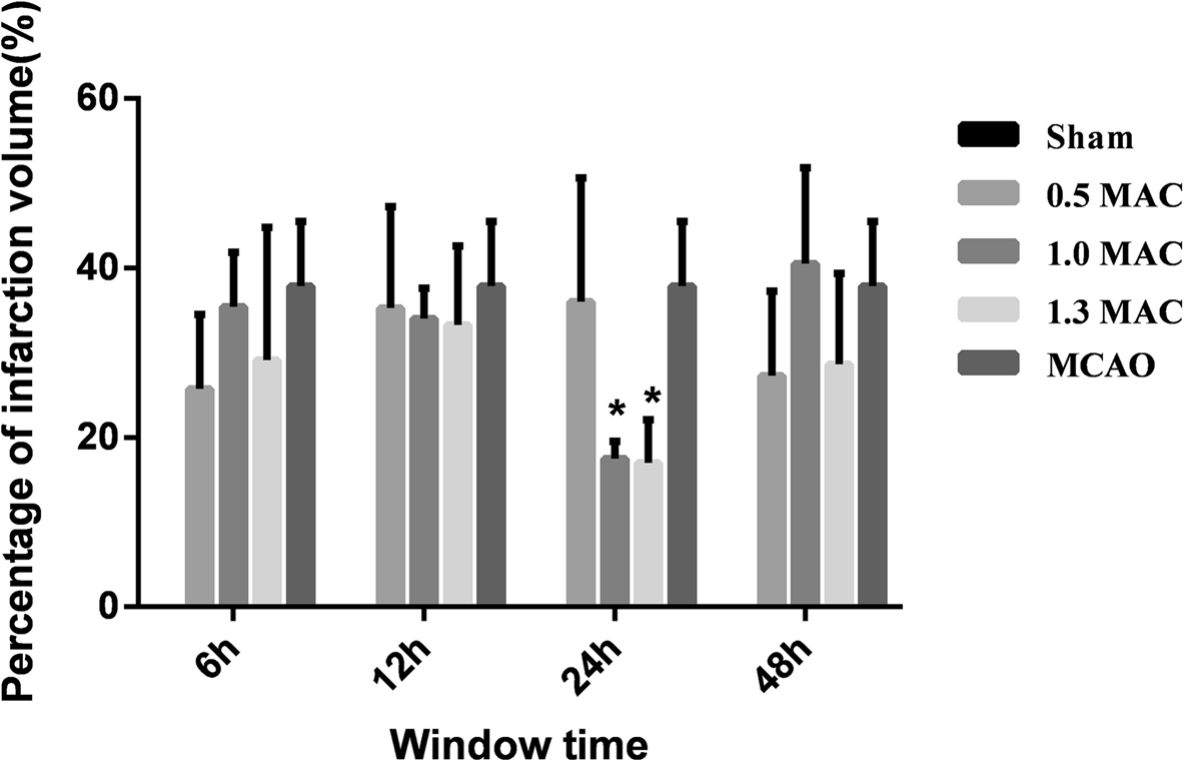
Comparison with MCAO group, the percentage of infarct volume was significantly lower in the 1.3 MAC sevoflurane preconditioning 24 h group. In 1.0 MAC sevoflurane preconditioning 24 h group, the cerebral infarct volume was significantly lower than the MCAO group. There was no statistical difference between other groups and MCAO group. Values are presented as mean ± SD, *n* = 5, *p < 0.05, vs. MCAO group

The percentage of infarct volume was 37.89 ± 7.66% in the MCAO group, 29.15 ± 15.68% in the 1.3 MAC sevoflurane preconditioning 6 h group, 33.29 ± 9.33% in the 12 h group, 17.01 ± 5.12% in the 24 h group, and 28.63 ± 10.73% in the 48 h group. Compared with the MCAO group, a statistically significant difference was observed with the 24 h group (*P* = 0.001), while no statistical difference was observed with the 6 h, 12 h, and 48 h groups (*P* = 0.152, 0.447, 0.154). The percentage of infarct volume was 35.4 ± 6.47% in the 1.0 MAC sevoflurane preconditioning 6 h group, 34.0 ± 3.61% in the 12 h group, 17.5 ± 2.08% in the 24 h group, and 40.5 ± 11.39% in the 48 h group. A statistically significant difference was observed in the 24 h group (*P* = 0.003), while no statistical difference in the 6 h, 12 h, and 48 h groups when compared with the MCAO group (*P* = 0.680, 0.581, 0.685). The percentage of infarct volume was 25.8 ± 8.73% in the 0.5 MAC sevoflurane preconditioning 6 h group, 35.3 ± 11.96% in the 12 h group, 36.0 ± 14.66% in the 24 h group, and 27.3 ± 9.95% in the 48 h group. Comparison with MCAO group showed no statistical difference with the 6 h, 12 h, 24 h, and 48 h groups (*P* = 0.064, 0.682, 0.754, 0.103), (Fig. 4).

### Determination results of brain water content

**Fig. 5:**
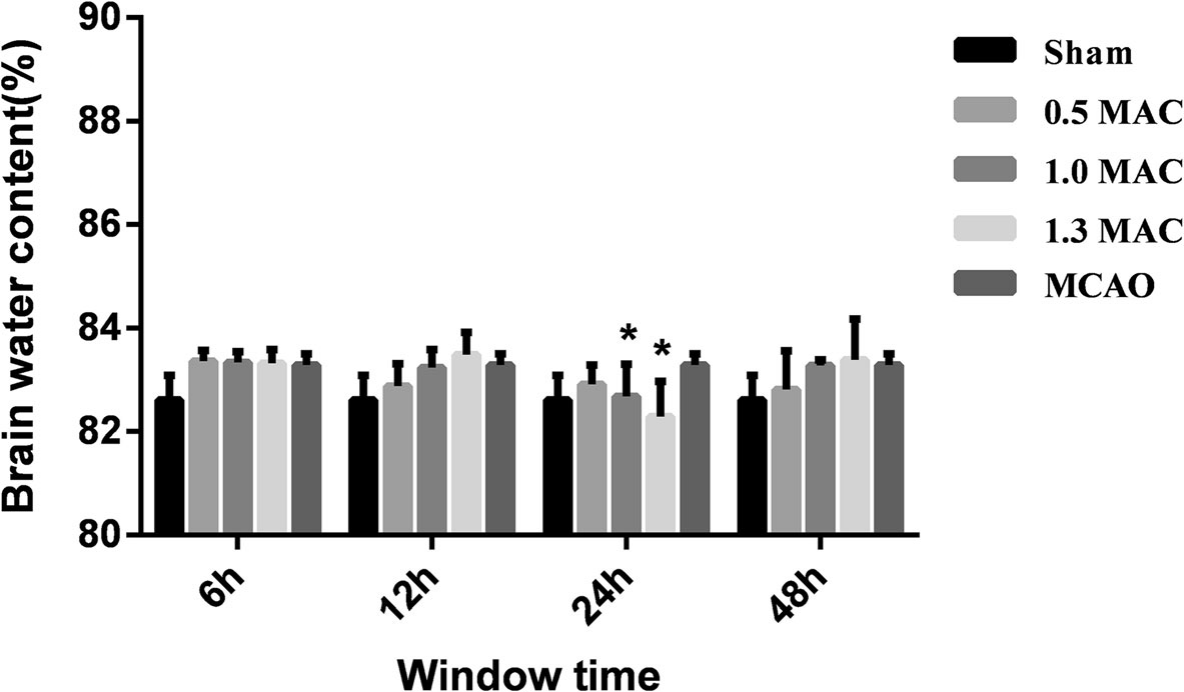
The brain water content in the 1.3 MAC sevoflurane preconditioning 24 h group and 1.0 MAC sevoflurane preconditioning 24 h group was significantly lower than the MCAO group. There was no statistical difference between others groups and the MCAO group. Values are presented as mean ± SD, *n* = 5, *p < 0.05, vs. MCAO group

The brain water content was 82.61 ± 0.48% in the sham group and 83.29 ± 0.21% in the MCAO group, and the difference was statistically significant (*P* = 0.014). The brain water content was 83.32 ± 0.27% in the 1.3 MAC sevoflurane preconditioning 6 h group, 83.49 ± 0.43% in the 12 h group, 82.29 ± 0.68% in the 24 h group, and 83.39 ± 0.79% in the 48 h group. Comparison with MCAO group showed a statistically significant difference with the 24 h group (*P* = 0.000), while no statistical significance was observed with the 6 h, 12 h, and 48 h groups (*P* = 0.905, 0.456, 0.911). The brain water content was 83.34 ± 0.21% in the 1.0 MAC sevoflurane preconditioning 6 h group, 83.24 ± 0.35% in the 12 h group, 82.68 ± 0.62% in the 24 h group, and 83.28 ± 0.11% in the 48 h group. Compared with the MCAO group, a statistically significant difference with the 24 h group (*P* = 0.027), and no statistical significance with the 6 h, 12 h, and 48 h groups (*P* = 0.870, 0.849, 0.977) were observed. The brain water content was 83.36 ± 0.21% in the 0.5 MAC sevoflurane preconditioning 6 h group, 82.88 ± 0.43% in the 12 h group, 82.91 ± 0.38% in the 24 h group, and 82.81 ± 0.57% in the 48 h group. There was no statistically significant difference with the 6 h, 12 h, 24 h, and 48 h groups when compared with the MCAO group (*P* = 0.812, 0.134, 0.160, 0.099), (Fig. 5).

## Discussion

This study used a single sevoflurane inhalation method to investigate whether preconditioning with different concentrations of sevoflurance can have brain protective effects. And the primary outcome is cerebral infarct volume. In the present study, after 1.3 MAC sevoflurane preconditioning, the MCAO model established after 24 h showed significant improving effects on the neurobehavioral score, cerebral infarct volume, and water content of brain tissue for the rats. This indicated that after 1.3 MAC sevoflurane preconditioning for 3 h, MCAO model established after 24 h showed effective brain protection. Compared with 0.5 MAC and 1.0 MAC preconditioning, the protective effects associated with 1.3 MAC sevoflurane preconditioning were more significant. This suggested that the brain protective effects of sevoflurane preconditioning were concentration depended in the literature [14]. With high concentrations of sevoflurane inhalation, the respiratory and circulatory systems can be inhibited in experimental animals [22], whereas no inhalation of higher concentration of sevoflurane for preconditioning was performed in the present study.

Preconditioning with 1.0 MAC sevoflurane for 3 h showed a significant improvement in the infarct volume and water content of brain tissues, but no significant improvement was observed in the behavioral score, which was not consistent with that of 2% sevoflurane inhalation that can effectively produce brain protective effects as reported in the literature. This might be due to differences in the preconditioning ways of sevoflurane [14, 19, 23]. Studies by Shiquan Wang et al. showed that 2% sevoflurane was inhaled each time for 1 h for 5 consecutive days for preconditioning, and MCAO model that was established after 24 h had effective neuroprotection [19]. According to a study by Ralphiel S et al., 30 min exposure to 1.0 MAC sevoflurane produces early neuroprotection against neuronal injury due to global cerebral ischemia induced by cardiac arrest. Repetitive 1.0 MAC sevoflurane anesthesia 30 min for 4 consecutive days conferred late neuroprotection effects against ischemic neuronal injury for 24 h preconditioning [23]. Studies by Qianzi Yang et al. found that 1, 2%, or 4% sevoflurane inhalation for 5 consecutive days indicated that sevoflurane preconditioning reduced infarct volume and improved neurobehavioral outcome in a dose-dependent manner by the MCAO model that was established after 24 h preconditioning. After preconditioning with 4% sevoflurane, the MCAO model was established had improved neurobehavioral score and showed significant cerebral infarct volume of rats [11].

In the present study, the time window of effective single preconditioning concentration of sevoflurane (1.0 MAC, 1.3 MAC) was 24 h, but the other time windows (6, 12, 48 h) after preconditioning showed no significant brain protective effects, which was consistent with the research results of Ralphiel S [23], Qianzi Yang [11], and Shiquan Wang [17] studies. However, some studies showed that if the MCAO model was established 50 min after inhalation of 1.0 MAC sevoflurane for preconditioning, 60 min sevoflurane preconditioning can induce the best neuroprotective effects in rats [24]. Another study also demonstrated that sevoflurane preconditioning can produce brain protective effects immediately after preconditioning [25]. The difference in time window of brain protection after this preconditioning was mainly due to the differences in concentration and time of sevoflurane preconditioning.

There are several mechanisms of brain protection for sevoflurane preconditioning, such as by inhibition of oxygen free radicals [11], anti-inflammation [15], activation of antioxidant enzymes [11], reduction of expression of apoptosis-related proteins [16], regulation of TREK-1 [9] and TREK-2 channel [8], inhibition of thioredoxin [17], etc. However, this study is limited as it considered only the concentration and time window of sevoflurane preconditioning, but the specific mechanism is not studied, which is also our future research direction.

## Conclusion

This study has investigated the appropriate concentration and time window for sevoflurane preconditioning. The results indicated that 1.3 MAC sevoflurane preconditioning for 3 h can be effective in reducing cerebral infarct volume and providing significantly cecebral protective effects. The optimal concentration was 1.3 MAC, and the optimal time window was 24 h after precondition.

## Data Availability

The data sets generated during the current study are available from the corresponding author on reasonable request.
